# Oncostatin M, A Profibrogenic Mediator Overexpressed in Non-Alcoholic Fatty Liver Disease, Stimulates Migration of Hepatic Myofibroblasts

**DOI:** 10.3390/cells9010028

**Published:** 2019-12-20

**Authors:** Beatrice Foglia, Salvatore Sutti, Dario Pedicini, Stefania Cannito, Claudia Bocca, Marina Maggiora, Maria Rosaria Bevacqua, Chiara Rosso, Elisabetta Bugianesi, Emanuele Albano, Erica Novo, Maurizio Parola

**Affiliations:** 1 Department Clinical and Biological Science, Unit of Experimental Medicine and Clinical Pathology, University of Torino, 10125 Torino, Italy; beatrice.foglia@unito.it (B.F.); stefania.cannito@unito.it (S.C.); claudia.bocca@unito.it (C.B.); marina.maggiora@unito.it (M.M.); mariarosaria.bevacqua@yahoo.it (M.R.B.); erica.novo@unito.it (E.N.); 2 Department of Health Science, University of East Piedmont, 28100 Novara, Italy; salvatore.sutti@med.uniupo.it (S.S.); emanuele.albano@med.uniupo.it (E.A.); 3 IRCC, Istituto per la Ricerca e la Cura del Cancro, Candiolo, 10060 Torino, Italy; dario.pedicini@hotmail.com; 4 Division of Gastroenterology and Hepatology, Department of Medical Science, University of Torino, 10154 Torino, Italy; chiara.rosso@unito.it (C.R.); elisabetta.bugianesi@unito.it (E.B.)

**Keywords:** Oncostatin M, liver fibrosis, NAFLD/NASH, myofibroblasts, migration, reactive oxygen species, VEGF-A

## Abstract

Background: Hepatic myofibroblasts (MFs) can originate from hepatic stellate cells, portal fibroblasts, or bone marrow-derived mesenchymal stem cells and can migrate towards the site of injury by aligning with nascent and established fibrotic septa in response to several mediators. Oncostatin M (OSM) is known to orchestrate hypoxia-modulated hepatic processes involving the hypoxia-inducible factor 1 (HIF-1). Methods. In vivo and in vitro experiments were performed to analyze the expression of OSM and OSM-receptor (OSMR) in three murine models of non-alcoholic-fatty liver disease (NAFLD) and -steatohepatitis (NASH) and in human NASH patients as well as the action of OSM on phenotypic responses of human MFs. Results: Hepatic OSM and OSMR levels were overexpressed in three murine NASH models and in NASH patients. OSM stimulates migration in human MFs by involving early intracellular ROS generation and activation of Ras/Erk, JNK1/2, PI3K/Akt as well as STAT1/STAT3 pathways and HIF-1α. OSM-dependent migration relies on a biphasic mechanism requiring early intracellular generation of reactive oxygen species (ROS) and late HIF1-dependent expression and release of VEGF. Conclusion: OSM is overexpressed in experimental and human progressive NAFLD and can act as a profibrogenic factor by directly stimulating migration of hepatic MFs.

## 1. Introduction

During chronic liver diseases (CLDs), liver fibrogenesis is sustained by an heterogeneous population of myofibroblast-like cells (MFs) that can originate mainly from hepatic stellate cells (HSC/MFs) and portal (myo)fibroblasts or, to a less extent, circulating and bone marrow-derived mesenchymal stem cell (MSCs) engrafting chronically injured liver [1,2,3,4,5,6]. At present, the involvement of epithelial to mesenchymal transition (EMT) as pro-fibrogenic mechanism leading to the origin of MFs from hepatocytes and/or cholangiocytes in progressive CLD is likely to be of minor relevance, as suggested by several elegant but mostly negative fate tracing studies [7,8,9,10,11]. In the scenario of pro-fibrogenic phenotypic responses of hepatic MFs, their migration towards the site of injury and their ability to align with nascent and established fibrotic septa in response to several stimuli (polypeptide mediators, hypoxia, reactive oxygen species or ROS) is considered as a relevant issue [4,12,13]. During CLD many peptide mediators, including platelet-derived growth factor (PDGF), monocyte chemoattractant protein-1 (MCP-1 or CCL2), angiotensin II (AT-II), vascular endothelial growth factor-A (VEGF-A) as well as ROS and/or hypoxic conditions, have been reported to stimulate HSC/MFs migration, then contributing to fibrosis progression [12,13,14,15,16,17]. Along these lines, OSM, a pleiotropic cytokine structurally and functionally related to the interleukin-6 (IL-6) cytokine family, has recently emerged as a mediator involved in CLDs progression [18,19,20,21,22,23,24]. OSM acts through the Janus kinase/signal transducer and activator of transcription (JAK/STAT) pathway and mitogen-activated protein kinases (MAPK) in order to critically regulate processes such as liver development and regeneration, hematopoiesis, and angiogenesis [23]. Remarkably, these processes are also regulated by hypoxia through the involvement of hypoxia-inducible factor 1 alpha (HIF1α). A previous study by Vollmer and colleagues [19] showed that HIF1α levels increased significantly after treatment of hepatocytes and hepatoma cells with OSM. These Authors also showed that HIF1α contributed to OSM-downstream signaling events, suggesting the existence of a cross-talk between OSM and hypoxia signaling in liver development and regeneration. Moreover, OSM is known to affect and regulate inflammatory response in several diseases affecting different organs and tissues [20]. Concerning the liver, in addition to the role in development and regeneration [23] as well as inflammatory response [20] OSM has been suggested to be involved also in the pathogenesis of steatosis and hepatic insulin resistance [21]. OSM is expressed in Kupffer cells even in normal liver but it is consistently over-expressed in cirrhotic livers [18]. OSM can signal through 2 different heterodimeric receptors that share gp130 (a common subunit receptor for ligands of IL-6 family) and involve either leukemia inhibitor factor receptor β (LIFRβ) or OSM receptor β (OSMRβ). In particular, LIFRβ is weakly expressed in normal livers, but more intensely in cirrhotic liver, in reactive ductules, bile ducts, colangiocytes, and perisinusoidal areas; OSMRβ is expressed at low level in hepatocytes of normal livers, but is was reported to be unchanged in cirrhotic samples. Moreover, in a study from the same research group [22] it was reported that OSM was able to modulate response of HSC/MFs by increasing collagen I and tissue inhibitor of metalloproteinase-1 (TIMP-1) secretion, suggesting a putative pro-fibrogenic role for OSM in CLD. More recently, a mechanistic experimental study has confirmed the ability of OSM to act as a pro-fibrogenic mediator by regulating both macrophage (resident and bone marrow-derived) and, as a consequence, MFs activation during the course of thioacetamide (TAA)-induced model of CLD [24]. These authors showed that OSM up-regulated the expression of pro-fibrogenic mediators such as TGF-β and PDGF in macrophages, suggesting that OSM may be indirectly pro-fibrogenic by up-regulating through TGF-β and PDGF, collagen and TIMP1 synthesis in HSC/MFs. In the mentioned study, liver fibrosis was significantly prevented in OSM knockout mice submitted to the TAA protocol vs related control littermates. Moreover, continuous expression of OSM in normal mouse liver, as forced by means of hydrodynamic tail vein injection (HTVi), resulted in severe fibrosis [24]. In the present study, we investigated the involvement of OSM in experimental and human NAFLD/NASH and we performed experiments in order to elucidate whether this cytokine may act directly on LX2 cells and primary culture of human HSC/MFs by regulating selected pro-fibrogenic phenotypic responses.

## 2. Materials and Methods

Materials: The methionine and choline-deficient (MCD) diet, the choline-deficient amino acid-refined (CDAA) diet, the high fat–high fructose diet (HFHF) as well as the related control diets, methionine choline supplemented (MCS) diet and choline-supplemented and amino acid-refined (CSAA) diet and normal (not supplemented with fat) diet were provided by Laboratorio Dottori Piccioni srl (Gessate, Milano, Italy). The kit for RNA retro-trascription and for real time PCR were purchased from Biorad (Berkely, CA, USA). Enhanced chemiluminescence (ECL) reagents, nitrocellulose membranes (Hybond-C extra) were from Amersham Pharmacia Biotech Inc. (Piscataway, NJ, USA). Human recombinant Oncostatin M (OSM) was from Petrotech (Rocky Hill, CT, USA). Monoclonal antibody against p-ERK1/2 (sc-7383), polyclonal antibody for p-STAT1 (sc-8394), STAT1 (sc-346), p-STAT3 (sc-8059), STAT3 (sc-7179), ERK1/2 (sc-292838), p-Akt1/2/3 (sc-7985-R), Akt1/2/3 (sc-8312), VEGF (sc-152), and HIF-1α (sc-10790) were from Santa Cruz Biotechnology (Santa Cruz, CA, USA). Polyclonal antibody for p-JNK and JNK1/2 were from Cell Signaling Technology (Danvers, MA, USA). PD98059, SP600125, LY294002 and apocynin were from Calbiochem (La Jolla, CA, USA). Monoclonal antibodies for β-actin, crystal violet, DCFH-DA and all other reagents of analytical grade as well as primers for RT-PCR were from Sigma Chemical Co (Sigma Aldrich Spa, Milan, Italy), Boyden’s chambers were from Neuro Probe, Inc. (Gaithersburg, MD, USA). The monoclonal neutralizing antibody against Flk-1 was obtained from ImClone (New York, NY, USA).

Methods: Animal experimentation. In this study we used three different dietary protocols for induction of progressive NAFLD that were carried out in 8 week old C57Bl/6 mice (Harlan Laboratories, Indianapolis, IN, USA) (*n* = 8 for any experimental group). Mice were fed as previously described [25] on the following dietary regimens: (i) Methionine and choline-deficient (MCD) diet or methionine and choline sufficient (MCS) control diet, (ii) choline-devoided and L-amino acid-defined (CDAA) diet or choline-sufficient L-amino acid-defined (CSSA), (iii) high fat–high fructose (HFHF) diet. Mice were then sacrificed at different experimental time points (4 days, 2, 4, and 8 weeks for MCD or MCS protocol, 12 and 24 weeks for CDAA or CSAA protocol, 24 weeks for HFHF and standard control diet). Mice were kept under specific pathogen-free conditions and maintained with free access to pellet food and water. Liver samples were obtained and immediately used/processed for morphological or molecular biology analyses or frozen and thereafter maintained at −80 °C for further analysis. The experiments complied with EU and national ethical guidelines for animal experimentation and all experimental protocols were approved by the Animal Ethic Committee of University of Oriental Piedmont, Novara, Italy and Italian Ministry of Health.

Human patients: The study on NASH patients was approved by the Ethics Committee of the Azienda Ospedaliera Universitaria Città della Salute (Turin, Italy). For this study we analyzed liver biopsies from NASH patients (*n* = 20) or from patients with simple steatosis (*n* = 10), referring to the Division of Gastroenterology and Hepatology of the University of Turin. All samples were collected at the time of first diagnosis; all subjects gave informed consent to the analysis, and the study protocol, which conformed to the ethical guidelines of the 1975 Declaration of Helsinki, was planned according to the guidelines of the local ethics committee.

Immunohistochemistry analysis: Liver sections from human patients with NASH or with simple steatosis were employed. Immunostaining procedure was as previously described [25]. Briefly, paraffin sections (2 μm thick), mounted on poli-l-lysine coated slides, were incubated with (i) the monoclonal antibody against OSM (Santa Cruz Biotechnology, Dallas, TX, USA; dilution 1:200) or (ii) the monoclonal antibody against human CD68 (Biorad, Hercules, CA, USA; dilution 1:80) or (iii) the secondary monoclonal antibody alone, as negative control. After blocking endogenous peroxidase activity with 3% hydrogen peroxide and performing microwave antigen retrieval in sodium citrate buffer pH6, primary antibodies were labeled by using EnVision, HRP-labeled System (DAKO) and visualized by 3′-diaminobenzidine substrate.

LX2 cells culture: Human LX2 cells, a model of immortalized and activated, MF-like, human HSC, originally kindly provided by Prof. Scott L. Friedman (Icahn School of Medicine, MS, USA), were cultured in Dulbecco’s modified Eagle’s medium (Sigma Aldrich Spa, Milan, Italy), supplemented with 10% fetal calf serum and 1% antibiotics. In most experiments we also used human HSCs (Clinisciences, Nanterre, France), were used between passages 4 and 7 when showing a phenotype of fully activated, MF-like HSCs (HSC/MFs), plated to obtain the desired sub-confluence level and then left for 24 h in serum-free Iscove’s medium to have cells at the lowest level of spontaneous proliferation [13]. LX2 cells or HSC/MFs were then exposed in culture conditions to human recombinant OSM 10 ng/mL for different times.

Cell migration and Chemotaxis: Non-oriented migration (chemokinesis) and chemotaxis of human LX2 (and HSC/MFs) were evaluated after exposure to PDGF-BB 10ng/mL, used as positive control, or to OSM 10 ng/mL, by performing the wound healing assay (20 h of incubation) or the modified Boyden’s chamber assay (6 h of incubation), as previously described [7,13]. For the wound healing assay LX2 or HSC/MFs cells were plated on collagen coated 24 wells (Falcon, Corning, NY, USA) and, were confluent, left for 24 h in their medium without serum to have cells at the lowest level of spontaneous proliferation. Then, a scratch on the cell monolayer was performed and the cells were exposed to medium with hrOSM (or where indicated with specific inhibitors) for 20 h, stained with crystal violet and finally observed at contrast phase microscope.

For the Boyden’s chamber assay, filter of 8 µm (Whatman Nuclepore™ track-etched polycarbonate membranes) were first coated with type I collagen (20 µg/mL) for 30 min at 37 °C.

2 × 10^4^ cells were placed in the upper compartment and were allowed to migrate through the pores of the filter into the lower compartment, in which chemotactic agents are present. At the end of incubation time (6 h), filters were removed from the Boyden’s chamber and stained with crystal violet. In some experiments, cells were pre-treated with specific inhibitors to check the relevance of specific proteins in process described.

Proliferation: Proliferation of human LX2 cells (and HSC/MFs) was evaluated by crystal violet proliferation assay by seeding cells in a 96-well plate at a density of 1.5 × 10^3^ cells per well. The cells were incubated in serum-free medium (SFM) for 24 h and then exposed to OSM 10 ng/mL. At the desired time, the medium was removed, and the cells were washed twice with phosphate-buffered saline, fixed with 11% glutaraldehyde; after fixation, cells were washed and then stained with 0.1% (*w*/*v*) crystal violet solution for 10 min. After washing with water, the crystal violet was solubilized with 50 μL of 10% acetic acid solution, and absorbance was measured at 595–650 nm using a microplate reader (SpectraMAX M3; Molecular Devices, Sunnyvale, CA, USA). In some experiments, BrdU incorporation assay was performed. LX2 cells or HSC/MFs were seeded in culture plates (1 × 10^4^ cells per well, 96 multiwell), for 24 h up to 72 h. The cell proliferation rate was evaluated by means of a colorimetric assay kit supplied by Roche Diagnostic (11647229001) according to manufacturer’s instructions.

Detection of intracellular generation of ROS. (A) DCFH-DA technique: Detection of ROS generation in cultured cells was performed by using the semi-quantitative DCFH-DA technique as previously described [7]. Cultured cells, seeded in 12-well culture plates (10^5^ cells/well), were exposed to OSM 10 ng/mL for 15 min or pre-treated with apocynin for 1h and then exposed with OSM 10 ng/mL. ROS generation was detected as the conversion of 2′,7′-dichlorodihydrofluorescein diacetate (DCFH-DA, 1 μM) into the corresponding fluorescent derivative. Cells were observed and photographed under a Leica fluorescence microscope (DMI 4000 B model). (B) combination of DCFH-DA technique and flow cytometric analysis: cells were seeded in P35 dishes (5 × 10^5^ cells/dish), cultured for 24 h and exposed to OSM alone or OSM plus 250 nM apocynin for 1 h before addition of 5 μM DCFH-DA (15 min of incubation in the dark). Cells were rapidly washed with PBS, collected by trypsinization, briefly centrifuged (1600 rpm for 5 min) and re-suspended in PBS for analysis. Detection of DCF green fluorescence (FL1) was performed on at least 5000 cells per sample with a FACScan equipped with a 488 nm argon laser using the CellQuest software (version 1.0.264.21, Becton-Dickinson, Milano, Italy). The peak of FL1 intensity of DCFH-DA-stained control cells grown without OSM was set to channel 101 and retained for all measurements [24]. As a positive control, cells were treated with 100 μM H_2_O_2_ for 15 min, stained with DCFH-DA as above and assayed during the same analytical session.

Western blot analysis: Total cell lysates, obtained as described [7,11,24], were subjected to sodium dodecyl sulfate-polyacrylamide gel-electrophoresis on 13.5%, 10%, or 7.5% acrylamide gels, incubated with desired primary antibodies, then with peroxidase-conjugated anti-mouse or anti-rabbit immunoglobulins in Tris-buffered saline-Tween containing 2% (*w*/*v*) non-fat dry milk and finally developed with the ECL reagents according to manufacturer’s instructions. Sample loading was evaluated by reblotting the same membrane with the un-phosphorylated form of protein or with β-actin antibody. In some experiments, protein levels in culture medium obtained from LX2 or HSC/MFs exposed to OSM for the desired time were evaluated by an immunoprecipitation procedure, as described previously [13].

Quantitative real-time PCR (Q-PCR): RNA extraction, complementary DNA synthesis, quantitative real-time PCR (Q-PCR) reactions were performed as previously described [6,10]. mRNA levels were measured by Q-PCR, using the SYBR^®^ green method as described [25]. The amplification mix was prepared using iTaq Universal Syber Green SuperMix (Biorad Laboratories, Berkeley, CA, USA) following manufacturer’s instructions and realtime PCR was performed using Miniopticon ThermoCycler Instrument (Biorad Laboratories, Berkeley, CA, USA). Oligonucleotide sequence of primers used for RT-PCR were:
**Primer****Sense****Reverse**murine OSMTTTCTCTGGGGATACCATCGGGAGACACGATGGGCTATGTmurine OSMβRGGAGACACGATGGGCTATGTCATCTGAGGTGATGGTGGTGmurine TBPCACATCACAGCTCCCCACCAAGCGGAGAAGATGCTGGAAAChuman OSMTACTGCTCACACAGAGGACGCCTATAGCCGCCATGCTCGChuman OSMRCGCGTCAGGTTTGCACTTA GTGTGTGGCACATTCCAAGhuman LIFRGGCTCATCACCACCTTCCAACCCCTTTCCCATCCCAACAAhuman HIF1αCCACCTATGACCTGCTTGGT TATCCAGGCTGTGTCGACTGhuman VEGFCCCACTGAGGAGTCCAACATTTTCTTGCGCTTTCGTTTTThuman CCL2 CCCCAGTCACCTGCTGTTAT AGATCTCCTTGGCCACAATGhuman IL6AGGAGACTTGCCTGGTGAAA CAGGGGTGGTTATTGCATCThuman TNF-αAACCTCCTCTCTGCCATCAA GGAAGACCCCTCCCAGATAGhuman GAPDHTGGTATCGTGGAGGACTCATGGAC ATGCCAGTGAGCTTCCCGTTCAGC 

TATA binding protein (TBP) and Gliceraldehyde-3-phosphate dehydrogenase (GAPDH) were used as internal reference for murine and human sample respectively, and co-amplified with target samples using identical Q-PCR conditions. Samples were run in triplicate and mRNA expression was generated for each sample. Specificity of the amplified PCR products was determined by melting curve analysis and confirmed by agarose gel electrophoresis.

Statistical analysis: Data in bar graphs represent means ± SEM, and were obtained from average data of at least three independent experiments. Luminograms and morphological images are representative of at least three experiments with similar results. Statistical analysis for these experiments was performed by Student’s t-test or ANOVA for analysis of variance when appropriate (*p* < 0.05 was considered significant).

## 3. Results

### 3.1. OSM and OSMR Expression in Three Different Models of Experimental NASH and in Human NASH Patients

**Figure 1:**
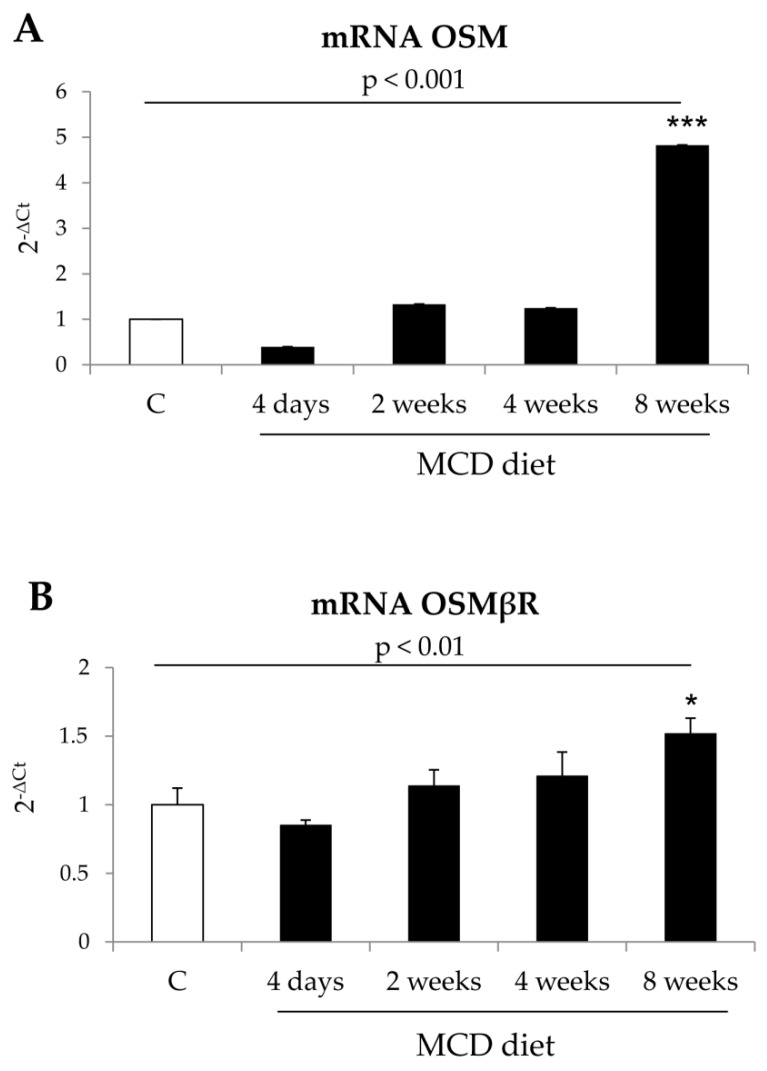

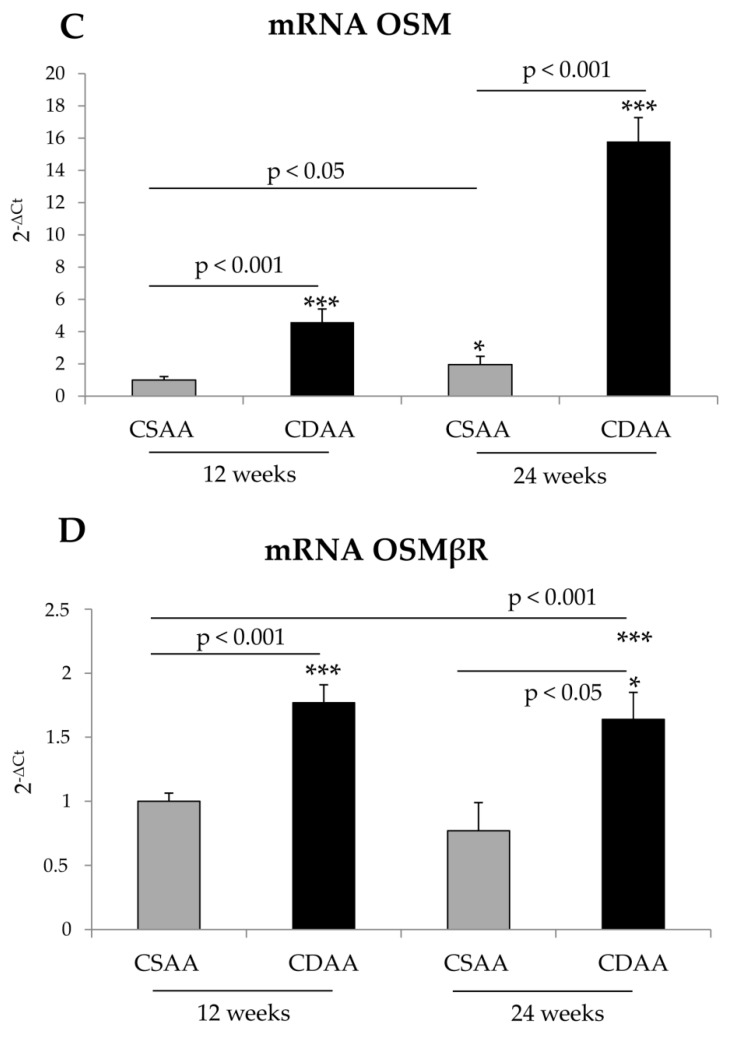

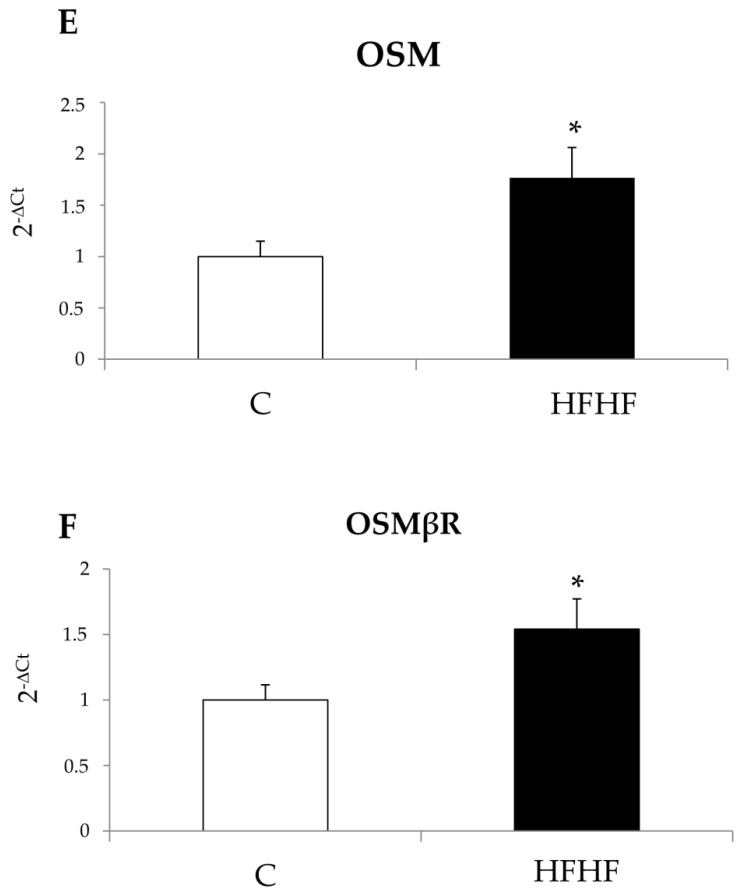
(**A**,**B**) Increased gene expression of Oncostatin M (OSM) and OSM receptor β (OSMRβ) in methionine and choline-deficient (MCD) mice model. Quantitative real time PCR (q-PCR) analysis of OSM (**A**) and OSMβR (**B**) transcripts levels in control mice (MCS diet) and in mice fed with MCD diet for 4 days, 2, 4, and 8 weeks. Data are expressed as means ± SEM of three independent experiments (*** *p* < 0.001, * *p* < 0.01 vs control mice.). (**C**,**D**) Increased gene expression of OSM and OSMβR in choline-deficient aminoacid-refined (CDAA) mice model. Quantitative real time PCR (q-PCR) analysis of OSM (**C**) and OSMβR (**D**) transcripts levels in control mice (CSAA diet) and in mice fed with CDAA diet for 12 and 24 weeks. Data are expressed as means ± SEM of three independent experiments (*** *p* < 0.001, * *p* < 0.05 vs control mice). (**E**,**F**). Increased gene expression of OSM and OSMβR in high fat–high fructose diet (HFHF) mice model. Quantitative real time PCR (q-PCR) analysis of OSM (**E**) and OSMβR (**F**) transcript levels in control mice (standard diet) and in mice fed with HFHF diet for 24 weeks. Data are expressed as means ± SEM of three independent experiments (* *p* < 0.05 vs control mice).

In order to investigate the involvement of OSM and its receptor OSMβR in NAFLD/NASH progression, mice were fed on MCD or CDAA or HFHF dietary protocols, all able to induce NAFLD progressing to NASH and liver fibrosis [25]. The results obtained showed that hepatic transcript levels for OSM and OSMβR significantly increased after 8 weeks of treatment in mice fed on the MCD protocol (Figure 1A,B) as well as in mice fed for 12 and 24 weeks on the CDAA protocol (Figure 1C,D) in parallel with increased collagen deposition and HSC/MF recruitment/activation, as previously described [25]. OSM and OSMβR transcript levels also increased in liver samples from mice fed on HFHF diet for 24 weeks (Figure 1E,F).

**Figure 2:**
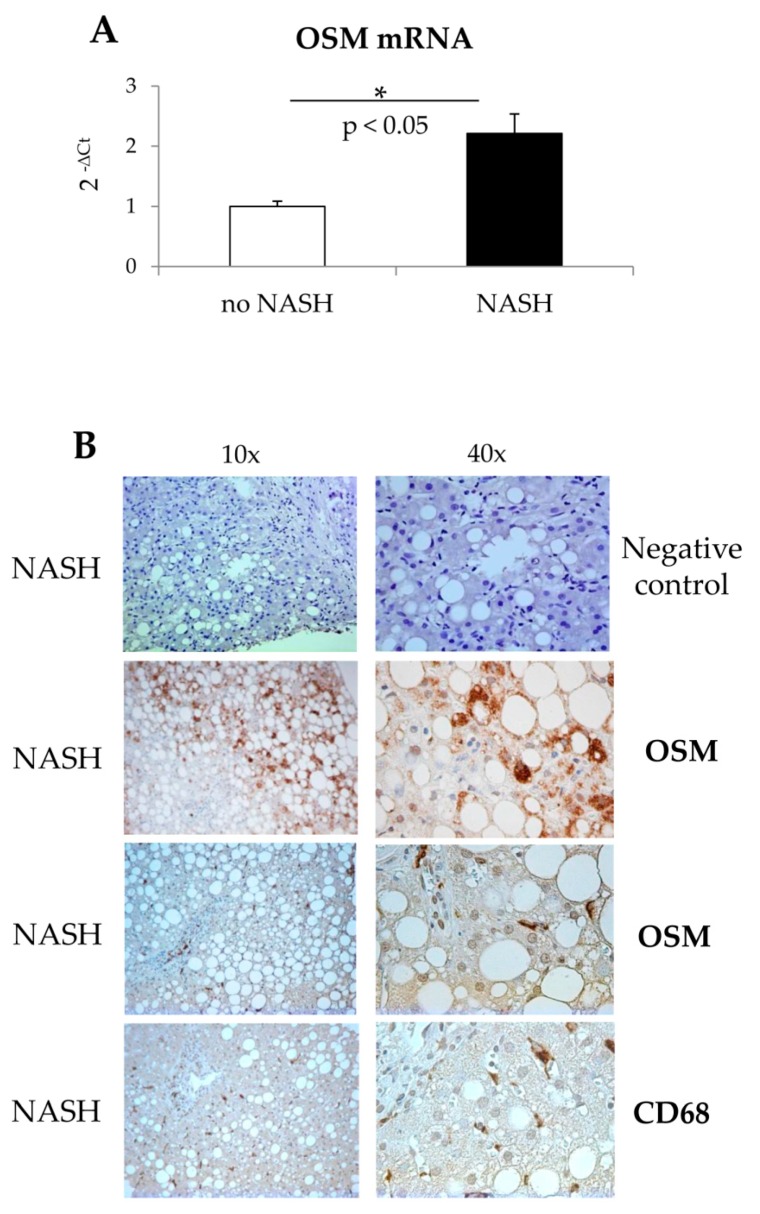
(**A**,**B**). Increased expression of OSM in human patients with NASH or with simple steatosis without NASH. (**A**) Quantitative real time PCR (q-PCR) analysis of OSM transcript levels in patients with NASH or with simple steatosis without NASH. Data are expressed as means ± SEM of three independent experiments (* *p* < 0.05 vs patients without NASH). (**B**) Immunohistochemistry analysis for OSM and CD68 (a marker of human macrophages) on liver specimens obtained from human patients with NASH. Original magnifications are indicated.

OSM transcript levels were significantly increased also in specimens obtained from NASH patients as compared to those obtained from patients showing simple steatosis (no NASH, Figure 2A); moreover, OSM immunostaining was also significantly increased in sections from liver biopsies from NASH patients and in cells characterized by macrophage-like morphology (i.e., CD68 positive) (Figure 2B).

### 3.2. OSM Effects on Profibrogenic Cells

**Figure 3:**
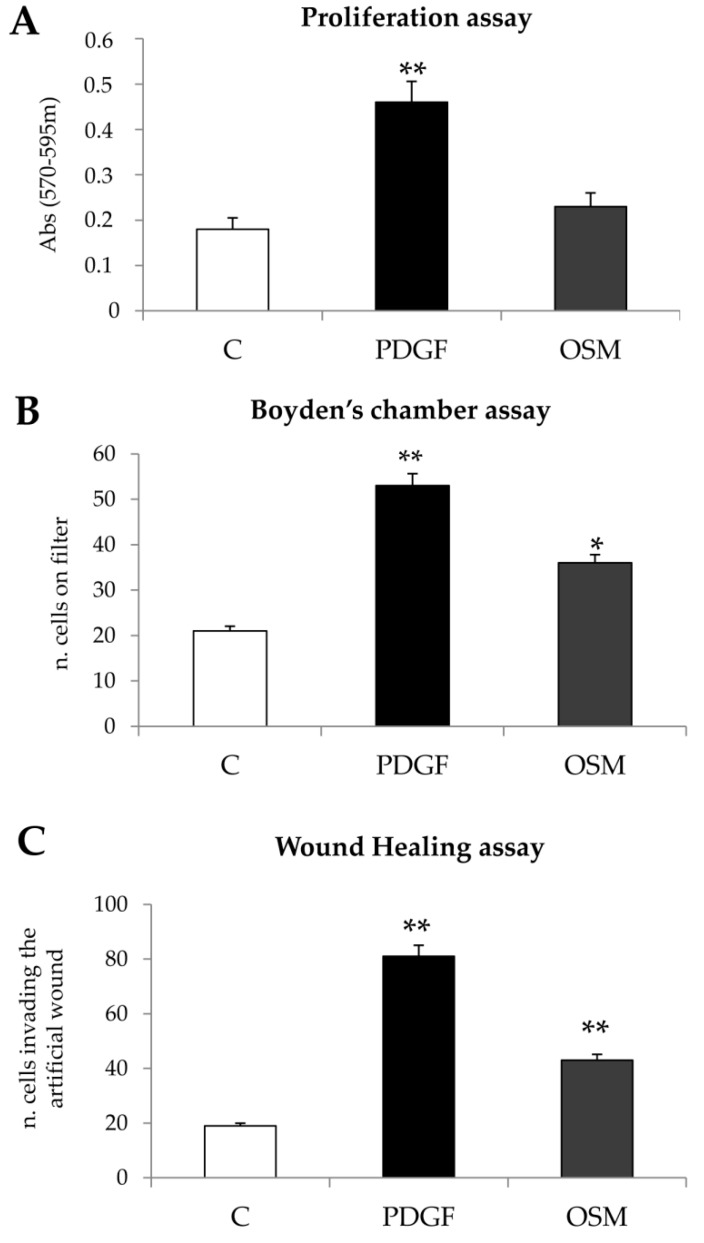
(**A**–**C**) Effect of human recombinant OSM (hrOSM) on proliferation and migration of LX2 cells. Proliferation assay (24 h, (**A**)), Boyden’s chamber assay (6 h, (**B**)) and wound healing assay (20 h, (**C)**) were performed on LX2 cells exposed to PDGF-BB 10 ng/mL, used as positive control, or to hrOSM 10 ng/mL. Data in bar graphs represent mean ± SEM (*n* = 3, in triplicate) * *p* < 0.05, ** *p* < 0.01 versus control value.

On the basis of results suggesting the involvement of OSM in the early stage of experimental NAFLD/NASH as well as in specimens from human NASH patients, we next analyzed the potential direct pro-fibrogenic role of OSM on myofibroblast-like cells. Human immortalized LX2 cells and, in parallel, human HSC/MFs in primary culture were exposed to human recombinant OSM (hrOSM) in order to evaluate selected pro-fibrogenic phenotypic responses of this cells. Human LX2 cells as well as HSC/MFs, that both positively responded to the potent mitogen and chemoattractant PDGF-BB (used here as positive control), did not proliferate when exposed to hrOSM according to data obtained in both the proliferation assay (Figure 3A and Figure S1A) and the BrdU incorporation assay (Figure S2A,B). However, hrOSM was able to stimulate chemotaxis and non-oriented migration in both LX2 cells (Figure 3B,C) and HSC/MFs (Figure S1B,C).

**Figure 4:**
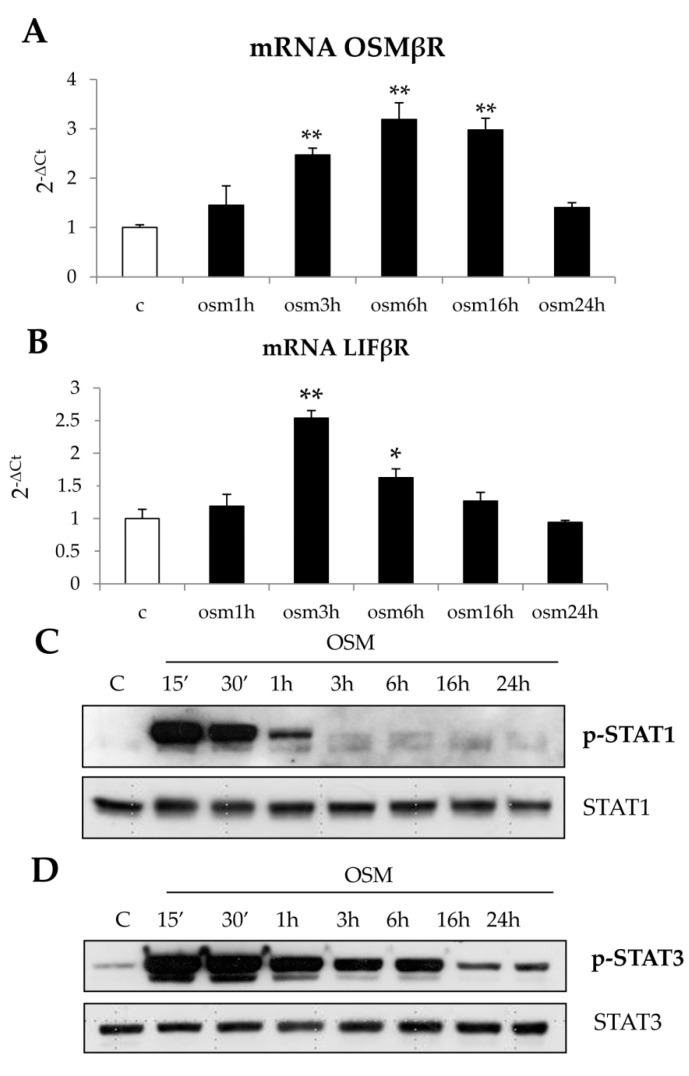
(**A**–**D**) Involvement of both OSM-receptors, OSMβR and LIFβR, as well as of STAT1 and STAT3 signal pathways in LX2 cells in response to hrOSM. Quantitative real time PCR (q-PCR) analysis of OSMβR (**A**) and LIFβR (**B**) transcript levels was performed in LX2 cells exposed to hrOSM 10 ng/mL up to 24 h. Data are expressed as means ± SEM of three independent experiments. * *p* < 0.05, ** *p* < 0.01 versus control value. Western blotting analysis of phosphorylated STAT1 (**C**) and STAT3 (**D**) in LX2 cells exposed to hrOSM 10 ng/mL (starting from 15 min up 12 h). Equal loading was confirmed by-reprobing the same membrane with the un-phosphorylated protein.

The response to hrOSM was based, as expected, on the expression of both LIFβR e OSMβR receptor subunits in cultured cells; interestingly, treatment of LX2 and HSC/MFs cells with hrOSM also determined a significant increase of mRNA levels for both receptors that was detected 3 h after OSM administration (Figure 4A,B and Figure S3A,B). hrOSM, according to literature data on other cell types, induced an increased, early and transient phosphorylation of STAT1, lasting from 15 min to 1 h (Figure 4C and Figure S3C) as well as an increased and more persistent (up to 6 h) phosphorylation of STAT3 (Figure 4D and Figure S3D).

**Figure 5:**
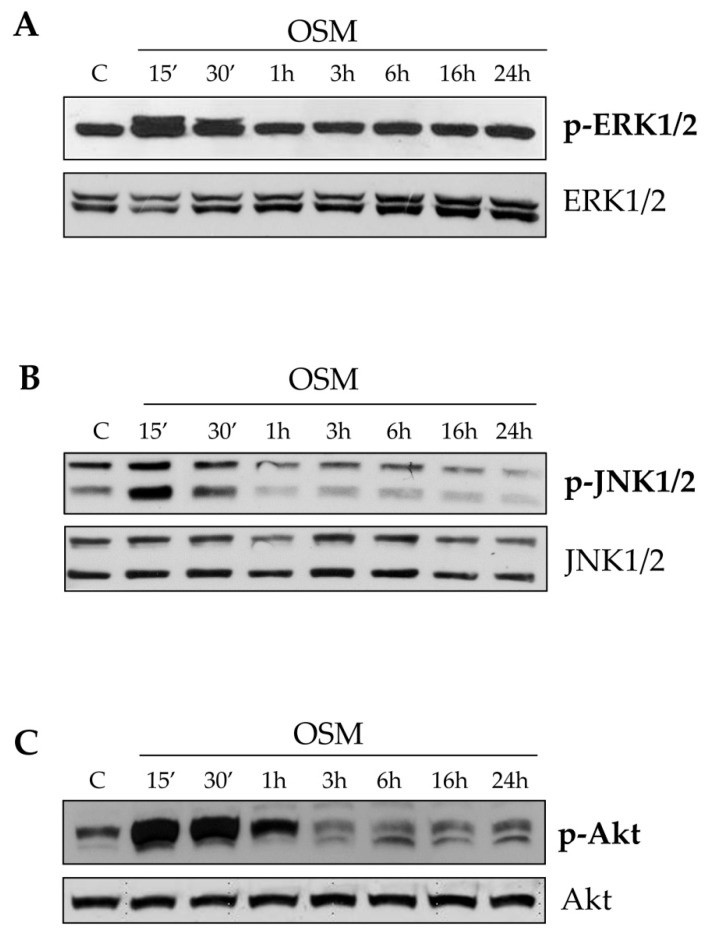
(**A–C**) The cellular response to hrOSM involved different signal pathways. Western blotting analysis of phosphorylated ERK 1/2 (**A**), JNK 1/2 (**B**) and c-Akt (**C**) in LX2 cells exposed to hrOSM 10 ng/mL (starting from 15 min up to 24 h). Equal loading was confirmed by reprobing the same membrane with the un-phosphorylated protein.

In both cell types OSM also induced an early (i.e., within 15–30 min) phosphorylation of ERK1/2 isoforms (Figure 5A and Figure S4A), JNK1/2 isoforms (Figure 5B and Figure S4B) and cAkt (Figure 5C and Figure S4C). As expected, the phosphorylation of all proteins analyzed did not change in control conditions at all experimental time points (Figure S5A,B).

**Figure 6:**
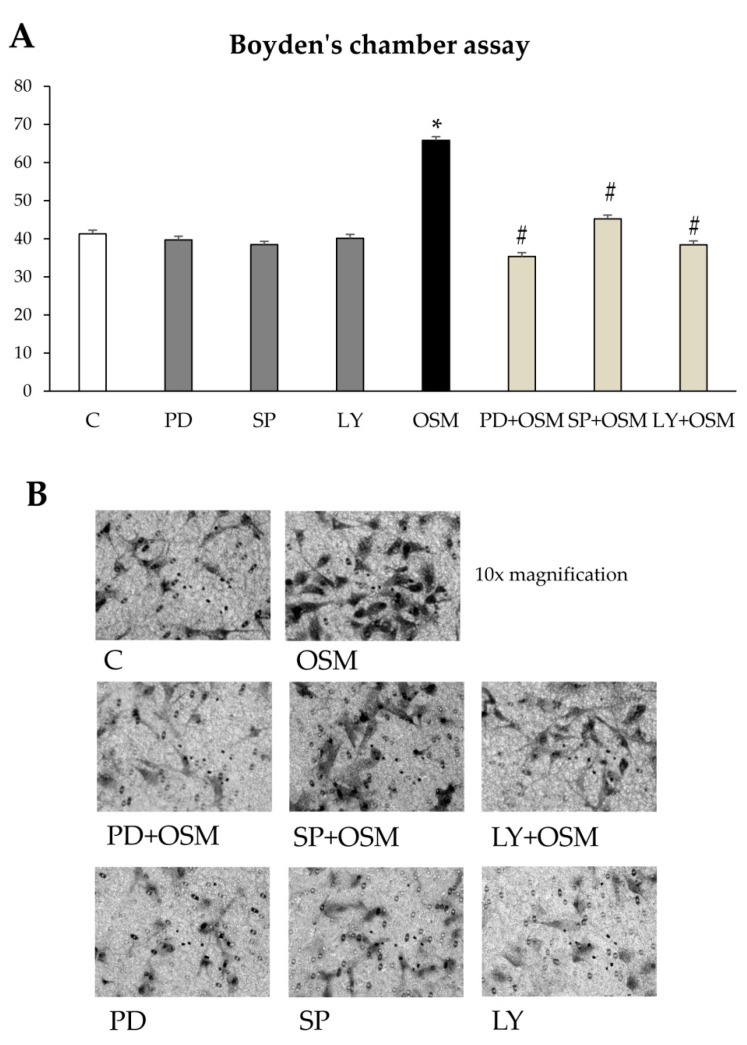

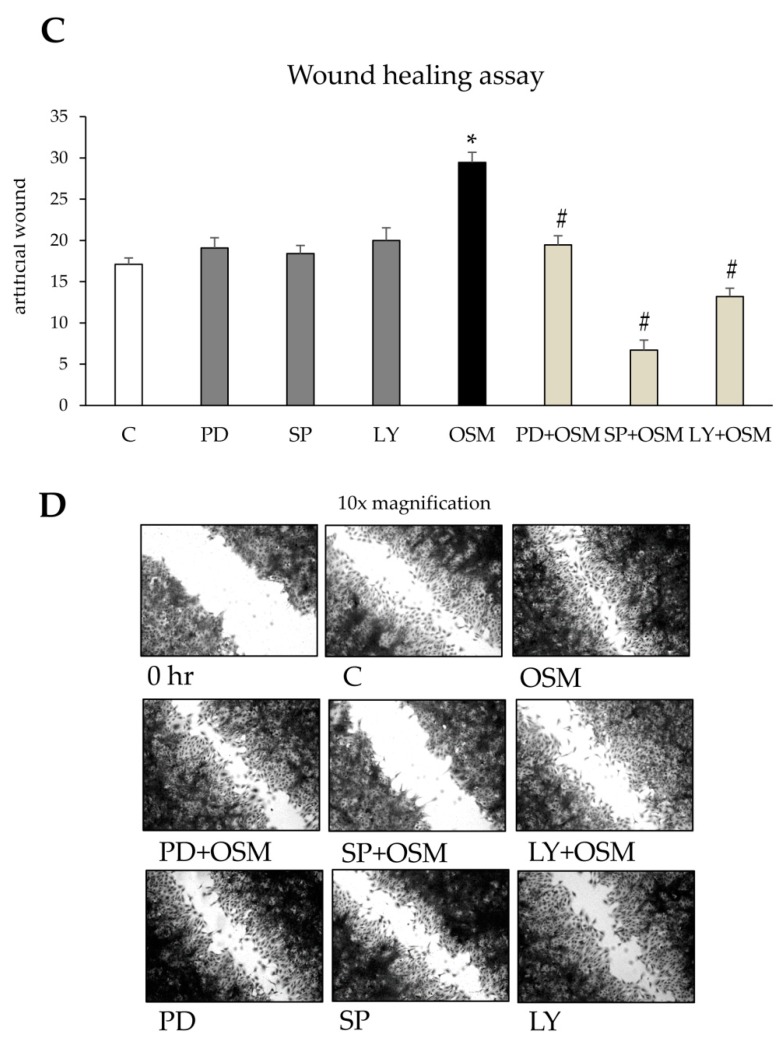
(**A**,**B**) Involvement of signal transduction pathways in the modulation of OSM-dependent migration of LX2 cells. Boyden’s chamber assay (**A**) was performed on LX2 cells exposed to hrOSM 10 ng/mL for 6 h. In some experimental conditions LX2 cells were pre-treated for 30 min with specific pharmacological inhibitors PD98059 (MEK-1 inhibitor), SP600125 (JNK 1/2 inhabitor), LY294002 (Akt inhibitor) and then exposed or not to hrOSM 10 ng/mL for 6 h. Data in bar graphs represent mean ± SEM (*n* = 3, in triplicate). * *p* < 0.05 versus control value; # *p* < 0.05, versus OSM value. (**B**) Representative images of cells on filter from Boyden’s chamber assay stained with crystal violet. Original magnification is indicated. (**C**,**D**) Involvement of signal transduction pathways in the modulation of OSM-dependent migration of LX2 cells. Wound healing assay (**C**) was performed on LX2 cells exposed to hrOSM 10 ng/mL for 20 h. In some experimental conditions LX2 cells were pre-treated for 30 min with specific pharmacological inhibitors PD98059 (MEK-1 inhibitor), SP600125 (JNK 1/2 inhibitor), LY294002 (Akt inhibitor) and then exposed or not to hrOSM 10 ng/mL for 20 h. Data in bar graphs represent mean ± SEM (*n* = 3, in triplicate). * *p* < 0.05 versus control value # *p* < 0.05, versus OSM value. (**D**) Representative images of cells invading the artificial wound stained with crystal violet. Original magnification is indicated.

In order to elucidate which signaling pathway had a key role in modulating cell migration, both cell types were pre-treated with specific pharmacological inhibitors. Pre-treatment of cells with PD98059 (inhibitor of the ERK1/2 upstream kinase MEK-1) or SP600125 (inhibitor of the JNK1/2) or LY294002 (inhibitor of the Akt upstream kinase PI3K) significantly reduced either chemotaxis (Figure 6A,B and Figure S6A,B) or chemokinesis (Figure 6C,D and Figure S6C,D) in both LX2 cells and HSC/MFs.

**Figure 7:**
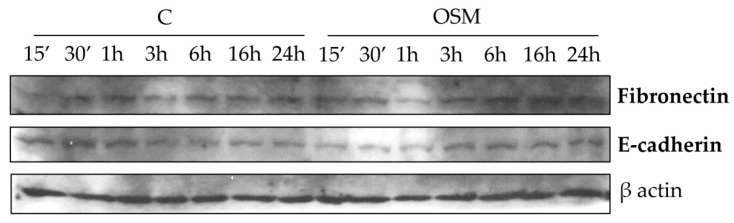
The effect of hrOSM in EMT process. Western blotting analysis of Fibronectin and E-cadherin in LX2 cells exposed or not (indicated as C) to hrOSM 10 ng/mL (starting from 15 min up to 24 h). Equal loading was confirmed by reprobing the same membrane with the β-action.

Since literature data reported a role of OSM in triggering epithelial mesenchymal transition (EMT), we next performed experiments to evaluate a possible role of OSM in inducing EMT markers. However, in both cell types OSM was not able to affect typical EMT markers, like fibronectin and E-cadherin (Figure 7 and Figure S7).

**Figure 8:**
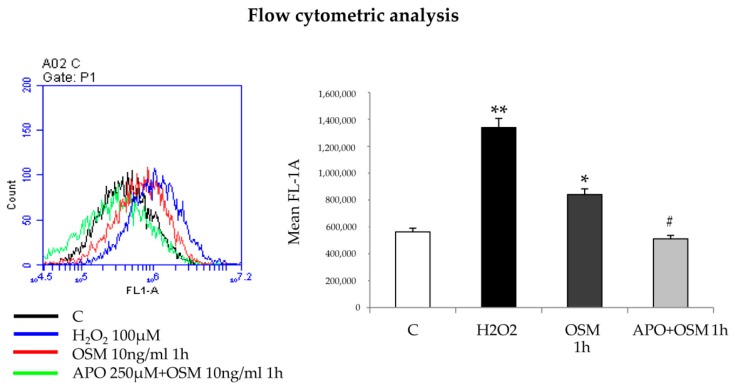
hrOSM dependent induction of migration relies on intracellular generation of ROS. Flow citometry analysis to evaluate ROS intracellular generation in LX2 cells untreated or treated for 1 h with hrOSM 10 ng/mL or with H_2_O_2_ 100 µM for 15 min, used as positive control, or pre-treated for 1 h with the pharmacological inhibitor of NADPH oxidase (APO: apocynin 250 µM) and then exposed to hrOSM 10 ng/mL for 1 h. * *p* < 0.05, ** *p* < 0.01 versus control value, # *p* < 0.05 versus OSM value.

In previous studies [12,13,26] we reported that exposure of human HSC/MFs to different polypeptides (PDGF, MCP-1, VEGF) and to controlled conditions of moderate hypoxia resulted in a significant increase of intracellular ROS levels. We then next performed experiments to investigate whether intracellular generation of ROS may be critical also after exposure of LX2 and HSC/MFs to hrOSM. In both cell types, OSM induced an early (within 1 h in LX2 cells and 15 min in HSC/MFs) and significant increase in ROS-related intracellular fluorescence, a finding that was prevented by pre-treating cells with the NADPH-oxidase pharmacological inhibitor apocynin (Figure 8 and Figure S8A), that although not specific, has the advantage to inhibit all NAPDH oxidase isoforms (NOX1, NOX2, and NOX4) expressed by hepatic MFs.

**Figure 9:**
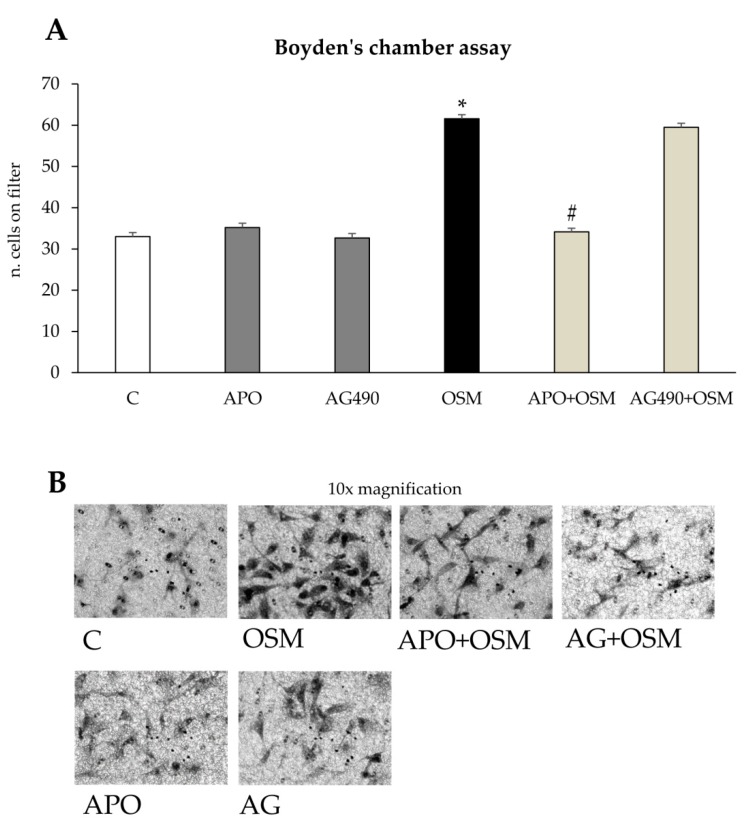

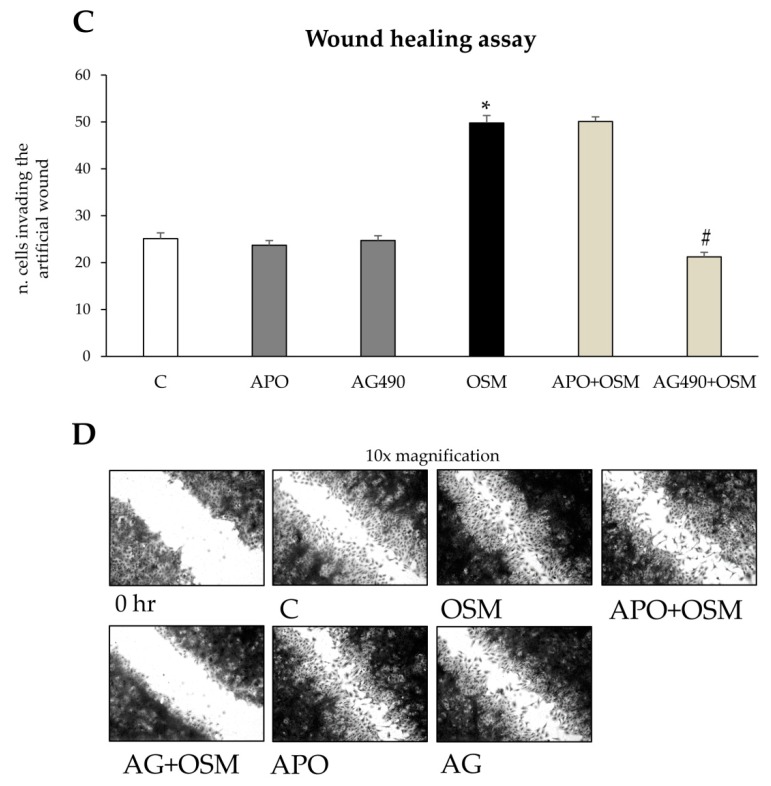
(**A**,**B**) OSM-dependent chemotaxis relies on ROS intracellular generation. Boyden’s chamber assay (**A**) was performed on LX2 cells treated with hrOSM 10 ng/mL for 6 h or, where indicated, pre-treated for 1 h with the pharmacological inhibitor of the NADPH oxidase (APO: apocynin 250 µM) or with the JAK2 inhibitor (AG490 100 µM) and then exposed or not to hrOSM 10 ng/mL for 6 h. * *p* < 0.05, versus control value, # *p* < 0.05 versus OSM value. (**B**) Representative images of cells on filter from Boyden’s chamber assay stained with crystal violet. Original magnification is indicated. (**C**,**D**) OSM-dependent non-oriented migration relies on STATI 1/3 activation. Wound healing assay (**C**) was performed on LX2 cells treated with hrOSM 10 ng/mL for 20 h or, where indicated, pre-treated for 1 h with the the pharmacological inhibitor of the NADPH oxidase (APO: apocynin 250 µM) or with the JAK2 inhibitor (AG490 100 µM) and then exposed or not to hrOSM 10 ng/mL for 20 h. * *p* < 0.05, versus control value, # *p* < 0.05 versus OSM value. (**D**) Representative images of cells invading the artificial wound stained with crystal violet. Original magnification is indicated.

Apocynin also significantly inhibited OSM-induced chemotaxis in LX2 (Figure 9A,B) and in HSC/MFs (Figure S8B,C), but was ineffective on OSM-dependent chemokinesis in both cell types (Figure 9C,D and Figure S8D,E). Since OSM also involved activation of STAT1/STAT3 pathway, we pre-treated both cell types with AG490, the pharmacological inhibitor of the STAT1/STAT3 upstream kinase Jak-2; the use of AG490 resulted in a significant inhibition of non-oriented migration (Figure 9C,D and Figure S8D,E) but did not apparently affect chemotaxis (Figure 9A,B and Figure S8B,C).

**Figure 10:**
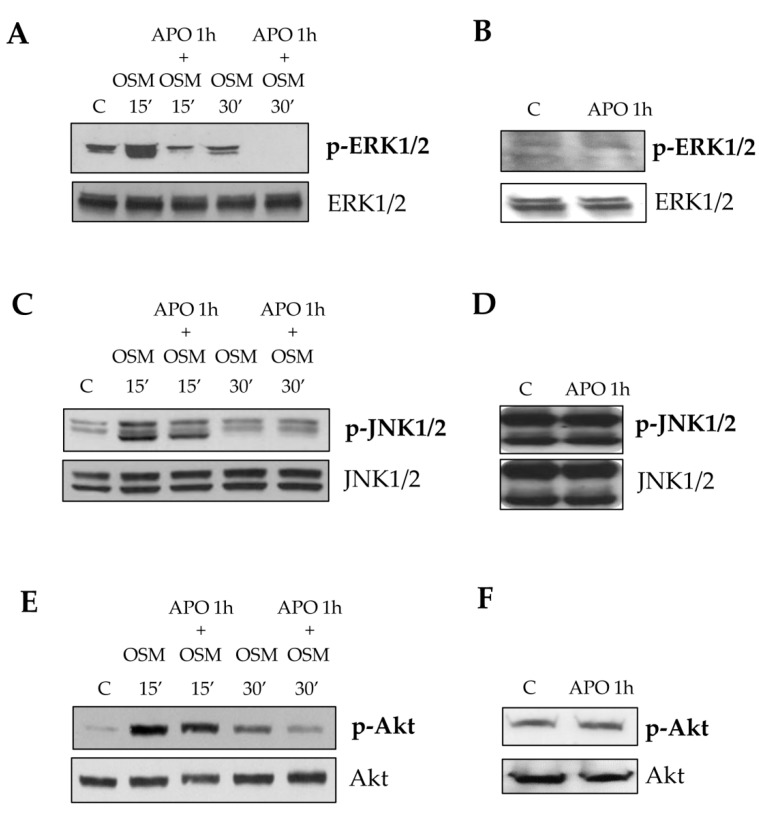
(**A**–**F**) Involvement of OSM-dependent intracellular ROS generation in the activation of specific signal pathways. Western blotting analysis of phosphorylated levels of ERK 1/2 (**A**,**B**), JNK 1/2 (**C**,**D**) and c-Akt (**E**,**F**) in LX2 cells exposed to hrOSM 10 ng/mL for 15 and 30 min or, where indicated, pre-treated 1 h with the pharmacological inhibitor of the NADPH oxidase (APO: apocynin 250 µM) and then exposed to hrOSM 10 ng/mL. In some experiments LX2 cells were treated with the inhibitor apocynin alone for 1 h (**B**,**D**,**F**) and incubated for additional 30 min. Equal loading was confirmed by re-probing the same membrane with un-phosphorylated protein.

In order to further elucidate the role of ROS in mediating the chemotactic response, we performed more experiments by pre-treating LX2 with apocynin. Apocynin significantly inhibited early phosphorylation of ERK1/2 isoforms, JNK1/2 isoforms and c-Akt (Figure 10A,C,E), confirming the critical role of intracellular ROS in mediating OSM-dependent chemotaxis of LX2, already described for other chemotactic peptides like PDGF-BB, CCL2 (MCP-1) and VEGF in HSC/MFs [13]. As expected, apocynin alone, did not affect the state of phosphorylation of ERK1/2, JNK1/2 as well as of c-Akt (Figure 10B,D,F).

**Figure 11:**
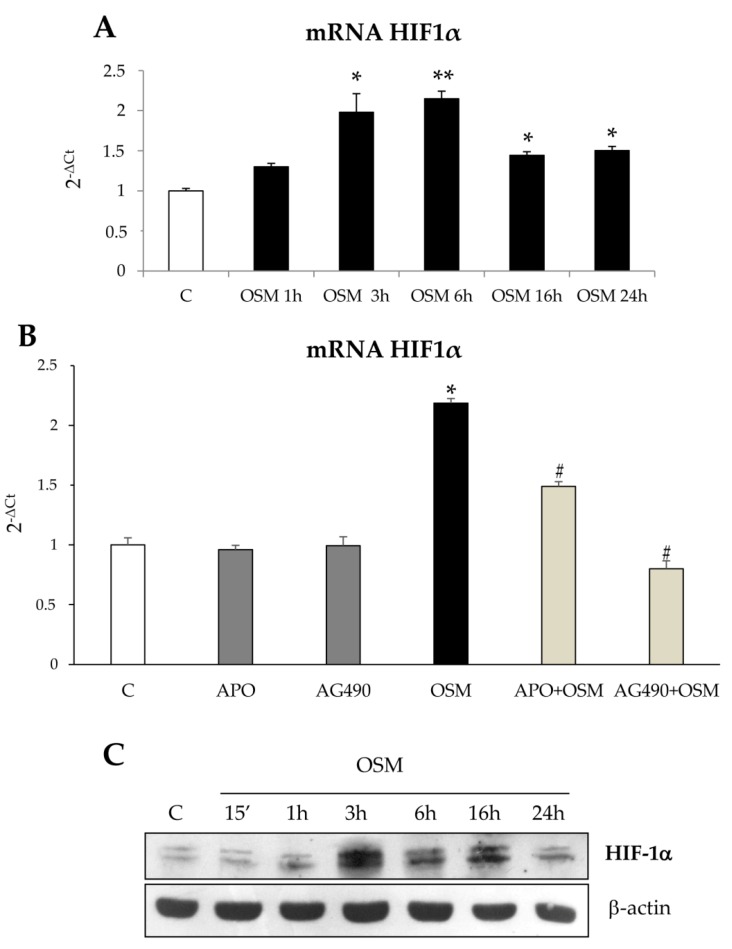
(**A**–**C**) Involvement of intracellular generation of ROS in the OSM-dependent recruitment/ stabilization of HIF1α. Quantitative real time PCR (q-PCR) analysis of HIF1α transcript levels in LX2 exposed to hrOSM 10 ng/mL up to 24 h (**A**) or in cells pre-treated with the pharmacological inhibitor of the NADPH oxidase (APO: apocynin 250 µM) or with the JAK2 inhibitor (AG490 100 µM) for 1 h and then exposed or not to OSM 10 ng/mL for 6 h (times in which HIF1α transcript levels were more significant). (**B**) Data are expressed as means ± SEM of three independent experiments. * *p* < 0.05, ** *p* < 0.01 versus control value, # *p* < 0.05; versus OSM value. (**C**) Western blotting analysis of HIF1α protein levels in LX2 untreated or treated with hrOSM 10 ng/mL (from 15 min to 24 h). Equal loading was confirmed by re-probing the same membrane with β-action.

Since in a previous study [13] we showed a peculiar biphasic nature of HSC/MFs migration in response to different polypeptides and controlled hypoxic conditions, we performed experiments to investigate whether the late non-oriented migration stimulated by OSM may depend on VEGF-A release. Moreover, since other researchers [19,20] proposed a role for OSM in orchestrating hypoxia-modulated hepatic processes involving the hypoxia inducible factor 1 (HIF-1), we performed experiments to evaluate whether OSM may contribute to HIF-1α recruitment/stabilization. In LX2 cells hrOSM induced an increase of HIF1α transcription (Figure 11A) which may depend on intracellular ROS production and JAK/STAT signaling, as suggested by inhibition observed in the presence of apocynin or AG490 (Figure 11B). Furthermore, the exposure of LX2 to rhOSM also induced an increase in HIF1α recruitment/stabilization; this event was significant after 3 h and remained significantly up-regulated until 24 h, although a decreasing trend was observed after 6 h (Figure 11C).

**Figure 12:**
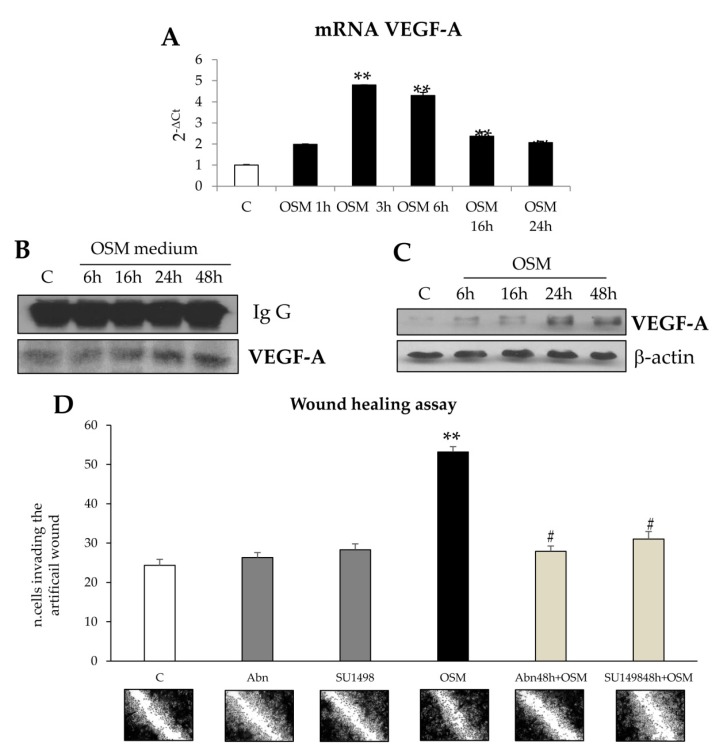
(**A**–**D**) Involvement of VEGF-A in OSM-dependent non oriented migration. (**A**) Quantitative real time PCR (q-PCR) analysis of VEGF-A transcript levels in LX2 exposed to hrOSM 10 ng/mL up to 24 h. Data are expressed as means ± SEM of three independent experiments. * *p* < 0.05, ** *p* < 0.01 versus control value. (**B**) Immunoprecipitation analysis of VEGF-A released in the medium of LX2 cells treated with hrOSM 10 ng/mL up to 48 h. (**C**)Western Blotting analysis of VEGF protein levels in LX2 cells treated with hrOSM 10 ng/mL up to 48 h. Equal loading was confirmed by re-probing the same membrane with p-actin. (**D**) Wound healing assay was performed in LX2 untreated or exposed to conditioned medium collected after 48 h of treatment with OSM (OSMm) for 20 h. In some experiments LX2 cells were pre-treated with the neutralizing antibody for VEGFR2 or with the pharmacological inhibitor of VEGFR2, SU1498, for l h and then exposed or not to OSMm for 20 h. ** *p* < 0.01 versus control value, # *p* < 0.05; versus OSMm value. Representative images of cells invading the artificial wound stained with crystal violet. Original magnification is indicated.

VEGF-A is a classic target gene of HIF1 (and then of HIF1α) and we previously demonstrated that this growth factor was able to stimulate migration of HSC/MFs [13]. Accordingly, we next investigated the involvement of VEGF-A in OSM dependent non-oriented migration of LX2 cells and HSC/MFs. Results indicate that exposure of LX2 cells to hrOSM increased both transcript (Figure 12A) and protein levels of VEGF-A (Figure 12C) and that these events occurred 24 h after hrOSM treatment, the time point at which VEGF-A is also released in the medium (Figure 12B). Homologous results were obtained also in HSC/MFs (Figure S9A–C).

In order to further investigate the relevance of OSM-dependent induction of VEGF-A expression, conditioned media obtained by cells exposed to hrOSM were collected after different time points (starting from 16 h to 48 h) and then used in the wound healing assay. OSM conditioned medium induced a significant stimulation of non-oriented migration at 48 h in both LX2 and HSC/MFs (Figure 12D and Figure S9D). Non-oriented migration was significantly inhibited by pre-treating cells with either a pharmacological inhibitor of VEGF receptor type 2 (VEGF-R2) tyrosine kinase (SU1498) or with a neutralizing antibody directed against VEGF-R2. As expected, apocynin alone did not affect basal non oriented migration (Figure 12D and Figure S9D). These data then suggest a pro-migratory role for VEGF-A released in the OSM conditioned medium (Figure 12D and Figure S9D).

**Figure 13:**
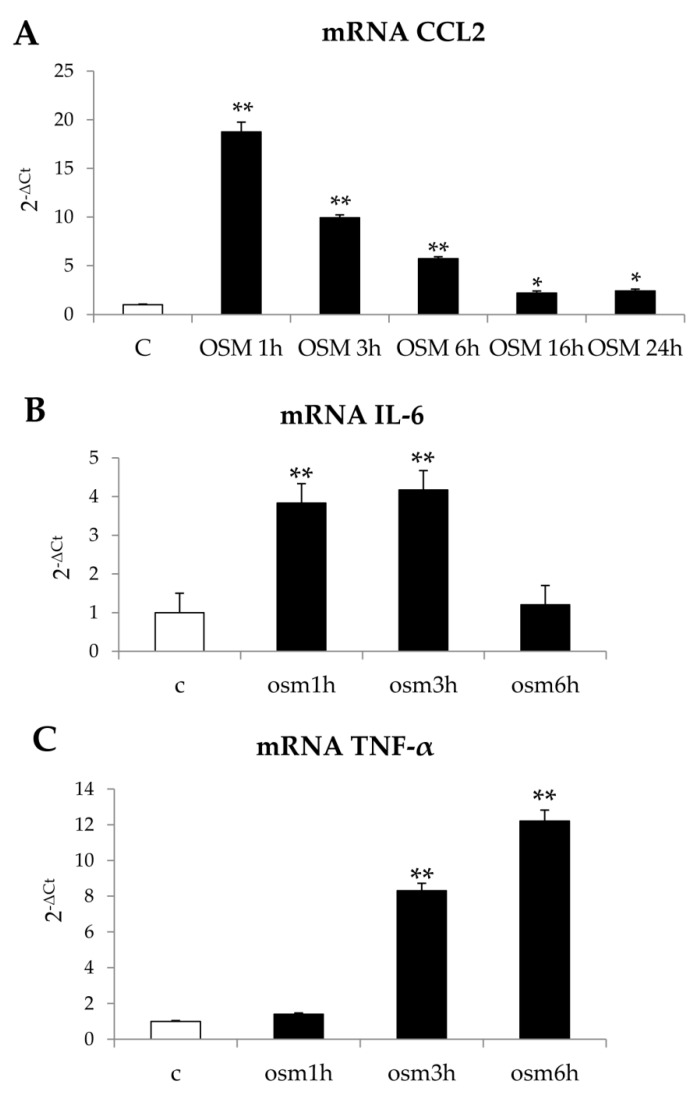
(**A**–**C**) OSM modulation of LX2 pro-inflammatory response. Quantitative real time PCR (q-PCR) analysis of CCL2 (**A**), TNF-α (**B**) and IL-6 (**C**) transcript levels in LX2 exposed to hrOSM 10 ng/mL for different experimental times. Data are expressed as means ± SEM of three independent experiments. * *p* < 0.05, ** *p* < 0.01 versus control value.

Finally, further preliminary experiments were performed in order to verify whether hrOSM, which is known to be mainly released by macrophages and lymphocytes, may also modulate the pro-inflammatory phenotypic response of LX2. The treatment of LX2 cells with hrOSM resulted in an increased transcription of CCL2, IL6, and TNFα (Figure 13A–C).

**Figure 14:**
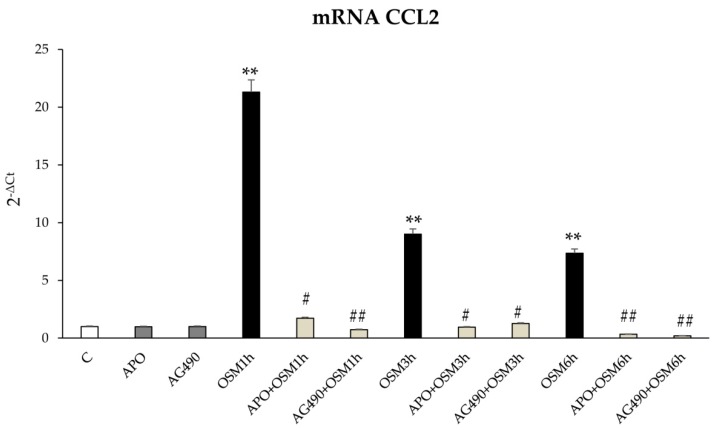
Intracellular ROS involvement in modulation of LX2 cells pro-inflammatory response. Quantitative real time PCR (q-PCR) analysis of CCL2 transcript levels in LX2 exposed to hrOSM 10 ng/mL for different experimental times. In some experimental conditions LX2 cells were pre-treated for l h with the pharmacological inhibitor of the NADPH oxidase (APO: apocynin 250 µM) or with the JAK2 inhibitor (AG490 100pM) and then exposed or not to hrOSM 10 ng/mL. Data are expressed as means ± SEM of three independent experiments. ** *p* < 0.01 versus control value, # *p* < 0.05, ## *p* < 0.01 versus OSM value.

Moreover, the pre-treatment of cells with apocynin or with AG490 reduced CCL2 transcription (Figure 14). The treatment with apocynin and AG490 did not affect the mRNA transcript levels of CCl2.

## 4. Discussion

Migration of hepatic MFs is a typical and distinctive pro-fibrogenic feature that allows these cells to align with inflammatory cells along fibrotic septa during liver fibrosis progression. This event occurs during CLDs in response to different stimuli and mediators, including polypeptide mediators, ROS as well as hypoxia [6,7,11]. In this scenario, literature data have already proposed a role for OSM in modulating CLD progression, likely by modulating defined processes like development, regeneration, hematopoiesis, and angiogenesis [20,22]. OSM is normally expressed in Kupffer cells and its expression, variable in normal livers, increases in cirrhotic livers [21]; it was proposed that this cytokine may up-regulate selected pro-fibrogenic responses of HSCs, including synthesis of collagen I and TIMP1 [22]. Along these lines, the present study was designed to investigate whether OSM may be involved also in NAFLD progression and may directly modulate other relevant pro-fibrogenic responses of MFs like proliferation and migration. Data in this study provide evidence that OSM is potentially involved in NAFLD progression as shown by data obtained by analyzing either the livers of mice fed on different dietary protocols (i.e., mice fed on either MCD, CDAA or HFHF diets) or in human liver specimens from NAFLD/NASH patients. In particular, human data indicate that OSM is up-regulated in NASH patients but not in patients that have only histological evidence of simple steatosis. Similarly, OSM expression increases in murine livers in parallel with the development of NASH-associated fibrosis. In our knowledge, this is the first report involving up-regulation of OSM expression in experimental and clinical progressive NAFLD, and further experiments and analyses are currently in progress in order to verify whether this mediator (or its related receptor subunits) may be validated as a marker of NAFLD progression and/or may have prognostic significance. This is a potentially relevant issue for a disease that is rapidly emerging as the most relevant CLD worldwide and that affects a large number of individuals in the general population.

Concerning the putative pro-fibrogenic role of OSM, already proposed by two earlier studies [18,22] and by a more mechanistic and recent one [24], we here propose that OSM is indeed able to directly target hepatic MFs. In particular, the present study shows that OSM can induce migration of both LX2 and HSC/MFs and that OSM dependent migration occurs as a biphasic process involving distinct but interrelated early and late events. These data are fully in agreement with previously published results investigating the pro-migratory action of chemotactic polypeptides and controlled conditions of hypoxia [12,13]. This conclusion is supported by several lines of evidence. First, OSM-induced early migration is triggered by ROS released within few minutes, likely as a consequence of the parallel activation of the ROS-generating membrane complex NADPH-oxidase, similarly to what described for HSC/MFs in response to other chemoattractant polypeptides [13]. The intracellular generation of ROS after OSM treatment was demonstrated by performing the DCFH-DA technique in presence or absence of the NADPH pharmacological inhibitor apocynin. This inhibitor, although not specific/selective, has the advantage to block all NADPH-oxidase (NOX) isoforms expressed by hepatic MFs (i.e., NOX1, NOX2, and NOX4). Apocynin pretreatment reduced early LX2 and HSC/MFs chemotaxis, whereas it was ineffective on late OSM-dependent migration. Our data also suggest, as previously demonstrated for other stimuli [13,25], that the rise of intracellular ROS generation is related to an early redox-dependent phosphorylation (i.e., activation) of ERK1/2 and JNK1/2 isoforms with a kinetic compatible with that of ROS release. This notion is supported by data demonstrating a reduction of chemotaxis in both cell types obtained by pre-treating cells with specific pharmacological inhibitors of ERK1/2 and JNK1/2 (PD98059 and SP600125, respectively). A direct link between the increase on intracellular levels of ROS and redox-related activation of both ERK1/2 and JNK1/2 it is now well established in the redox signaling scenario. It has been suggested that the increased kinase activity is likely to be the result of direct activation of defined signaling components or of inhibition of phosphatases responsible for the negative feed-back control of activated signaling pathways (like JNK specific phosphatases or, more generally, protein tyrosine phosphatases) [26,27,28]. The involvement of these different signaling pathways is in line with that observed for all the cytokine belonging to IL-6 type family, even if the signal transduction pathways of these cytokines, still currently investigated, are sometimes suggested to be cell-type specific.

A second role for OSM-related increase in intracellular ROS levels is related to the modulation of JAK/STAT signaling pathway. This event has been already described by Madamanchi et al. in vascular smooth muscle cells in which JAK2 was rapidly activated after treatment with hydrogen peroxide and both STAT1 and STAT3 were phosphorylated on tyrosine residues to then translocate to the nucleus in a JAK2-dependent manner [29]. Moreover, Authors showed that the inhibition of JAK2 activity by means of AG-490 partially inhibited hydrogen peroxide-induced ERK2 activity, suggesting that JAK protein is upstream of the Ras/ERK pathway and have a key role in the adaptive response to oxidative stress [29]. Along these lines, also in LX2 and HSC/MFs the early intracellular generation of ROS is able to sustain late migration by the involvement of JAK/STAT3 signaling pathway; this notion is supported by results showing AG490-dependent inhibition of JAK pathway and apocynin-dependent inhibition of STAT3 pathway. These results suggest that OSM can affect cell migration and are in agreement with a study in which the inhibition of JAK family members, in particular of JAK1, significantly reduced activation, proliferation, and migration of rat hepatic stellate cells [29]. Our results are also in agreement with literature data showing a role for JAK/STAT pathway in the modulation of liver fibrosis, as in the case of STAT3 activation in hepatic stellate cells treated with IL-6 and leptin [30,31].

Results obtained in the wound healing assay also provided evidence that OSM-dependent non oriented migration required late HIF1-dependent expression and release of VEGF-A. These results are homologous to those reported for exposure to hypoxic conditions and are potentially in agreement with literature data showing a role for OSM in modulating hypoxia-regulated processes (including hematopoiesis, angiogenesis, liver development, and regeneration), then suggesting a possible cross-talk between OSM and hypoxia signaling pathways [23]. According to literature data obtained in different cell types [19], we report that in both LX2 cells and HSC/MFs OSM can induce a significant up-regulation of HIF1α protein levels under normoxic conditions. Interestingly, as previously reported for HSC/MFs exposed to hypoxia [26], HIF1α up-regulation was associated with increased production and release of VEGF-A that, in turn, was the real responsible for late OSM-dependent migration. This notion was supported by the following observations: (i) OSM-related increase in HIF1α stabilization was rapidly detected (starting from 3 h and persisting for the entire experimental protocol; (ii) VEGF-A was released in the extracellular medium of cells exposed to OSM only after 24 h in LX2 cells; (iii) conditioned medium obtained from cells treated with OSM efficiently stimulated only late non-oriented migration; and (iv) both pre-treatment of cells with SU1498, a pharmacological inhibitor of the tyrosine-kinase receptor subunit of VEGFR-2 and with a neutralizing antibody for VEGFR-2 inhibited non-oriented OSM-dependent migration. These results obtained in both LX2 cells and HSC/MFs are in agreement with data obtained in previous studies related to the pro-migratory action of VEGF-A [26,32], and suggest a potential role for OSM-dependent expression and release of VEGF-A.

Finally, although in our experimental conditions OSM was found ineffective on the synthesis of collagen I and of TIMP1 (data not shown), differently from what reported by others [33], we additionally observed that hrOSM up-regulated in LX2 cells transcript levels of the chemokine CCL2, the most relevant chemokine during both experimental and human conditions of progressive NAFLD [34], as well as of the pro-inflammatory cytokines TNF e IL-6. However, these data are preliminary and further experiments will be necessary to mechanistically characterize this issue.

In conclusion, data reported in the present study provide novel evidence adding further knowledge on the proposed pro-fibrogenic role of OSM in the progression of CLD and suggest for the first time a possible involvement of this cytokine in the progression of NAFLD.

## Supplementary Materials

The following are available online at https://www.mdpi.com/2073-4409/9/1/28/s1, Figures S1–S6.
