# Supplementary material for: Oncostatin M, A Profibrogenic Mediator Overexpressed in Non-Alcoholic Fatty Liver Disease, Stimulates Migration of Hepatic Myofibroblasts
Source: Cells. 2019 Dec 20;9(1):28. doi: 10.3390/cells9010028 (PMC7017087; doi:10.3390/cells9010028)
Supplement: Supplementary file 1 [file cells-09-00028-s001.pdf]

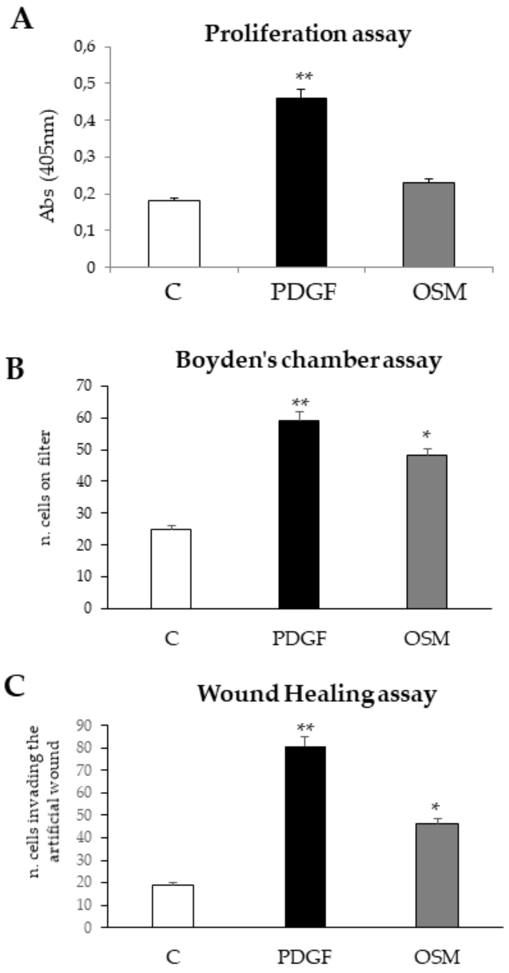

Figure 1. (**A**–**C**) Effect of hrOSM on proliferation and migration of HSC/MFs cells. Proliferation assay (**A**) Boyden's chamber assay (**B**) and wound healing assay (**C**) were performed on HSC/MFs exposed to PDGF-BB 10 ng/mL, used as positive control, or to hsOSM 10 ng/mL. Data in bar graphs represent mean ±SEM ($n = 3$, in triplicate). *$p < 0.05$, **$p < 0.01$ versus control value.

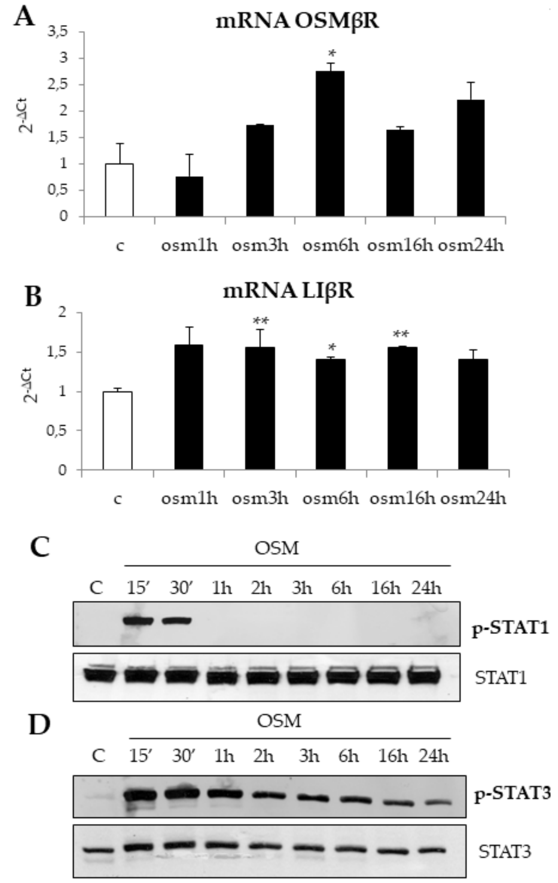

Figure 2. (**A**–**D**) Involvement of both OSM-receptors, OSMβR and LIFβR, as well as of STAT1 and STAT3 signal pathways in HSC/MFs in response to hrOSM. Quantitative real time PCR (q-PCR) analysis of OSMβR (**A**) and LIFβR (**B**) was performed in HSC/MFs exposed to hrOSM 10 ng/mL up to 24 h. Data are expressed as means ± SEM of three independent experiments. **p* < 0.05, versus control value. Western blotting analysis of phosphorylated STAT1 (**C**) and STAT3 (**D**) in HSC/MFs exposed to hrOSM 10 ng/mL (starting from 15 min up to 24 h). Equal loading was confirmed by reprobing the same membrane with un-phosphorylated protein.

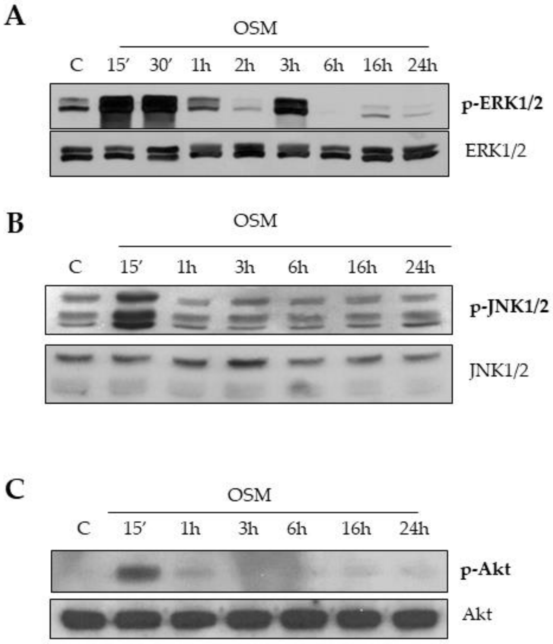

Figure 3. (**A**–**C**) HSC/MFs response to hrOSM involved different signal pathways. Western blotting analysis of phosphorylated ERK 1/2 (**A**), JNK1/2 (**B**) and c-Akt (**C**) in HSC/MFs exposed to hrOSM 10 ng/mL (starting from 15 min up to 24 h). Equal loading was confirmed by reprobing the same membrane with the un-phosphorylated protein.

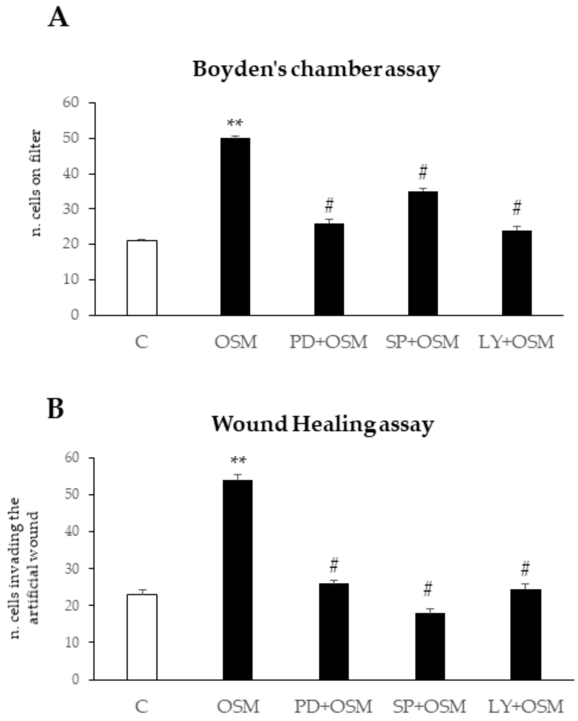

Figure S4. (**A**,**B**) Involvement of signal transduction pathways in the modulation of OSM-dependent migration of HSC/MFs. Boyden's chamber assay (**A**) and wound healing assay (**B**) were performed on HSC/MFs treated with hrOSM 10 ng/mL or ,where indicated, pre-treated for 30 min with specific pharmacological inhibitors PD98059 (MEK-1 inhibitor), SP600125 (JNK 1/2 inhibitor), LY294002 (Akt inhibitor) and then exposed to hrOSM 10 ng/mL. ***p* < 0.01 versus control value,#*p* < 0.05 versus OSM value.

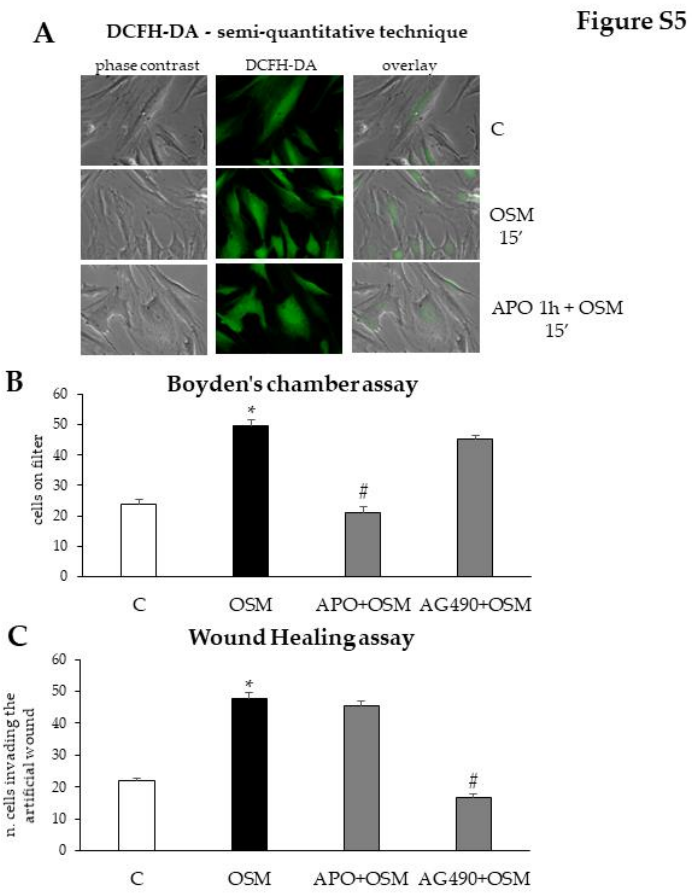

Figure 5. (**A**–**C**) hrOSM dependent induction of migration relies on intracellular genereation of ROS and STAT1/3 activation. DCFH-DA semiquatintiative analysis was performed to evaluate ROS intracellular generation in HSC/MFs untreated for 15 min with OSM 10 ng/mL or pre-treated for 1 h with apocynim, the pharmacological inhibitor of NADPH oxidase (APO 250 μM), and then exposed to OSM 10 ng/mL for 1 h (**A**). Boyden's chamber assay (**A**) and wound healing assay (**B**) were performed on HSC/MFs cells treated with hrOSM 10 ng/mL or ,where indicated, pre-treated for 1 h with apocynin, the pharmacological inhibitor of the NADPH oxidase (APO 250 μM), or with the JAK2 inhibitor (AG490 100 μM) and then exposed to OSM 10 ng/mL. *$p < 0.05$, versus control value, #$p < 0.01$ versus OSM value.

Figure S6

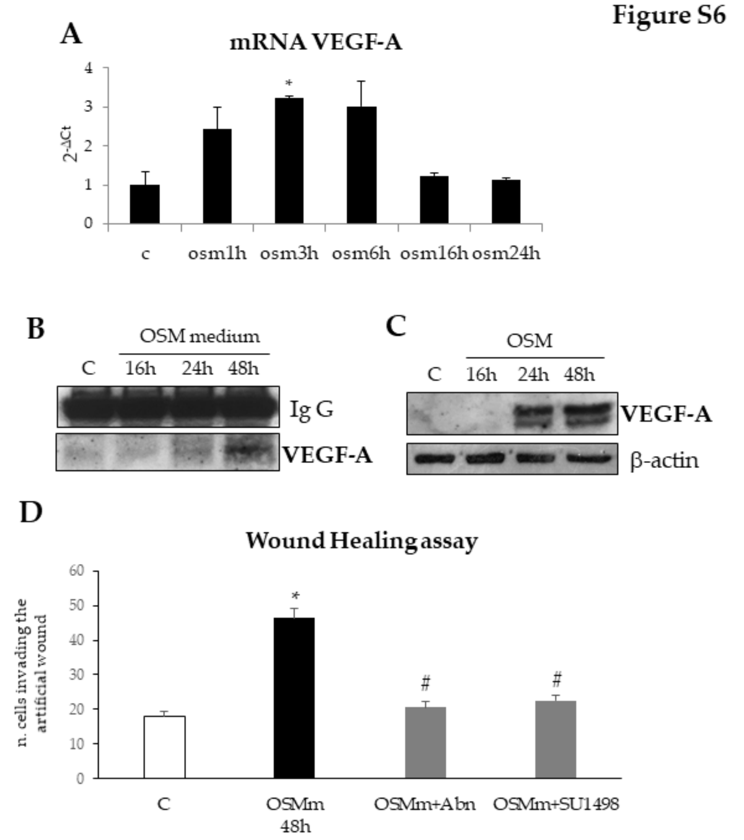

Figure 6. (**A**–**D**) involvement of VEGF-A in OSM-dependent non oriented migration. Quantitative real time PCR (q-PCR) analysis of VEGF-A (**A**) in HSC/MFs exposed to hrOSM 10 ng/mL up to 24 h. Data are expressed as means ±SEM of three independent experiments. *$p < 0.05$, **$p < 0.01$ versus control value. Immunoprecipitation analysis of VEGF-A released in the medium of HSC/MFs treated with hrOSM 10 ng/mL from 16 to 48 h (**B**). Western blotting analysis of VEGF protein levels in HSC/MFs treated with hrOSM 10 ng/mL from 16 to 48 h (**C**). Equal loading was confirmed by re-probing the same membrane with β-action. Wound healing assay (**D**) in HSC/MFs untreated or exposed to medium (OSMm) obtained from HSC/MFs treated with OSM for 48 h or, where indicated, pre-treated with the neutralizing antibody for VEGFR2 or with the pharmacological inhibitro of VEGFR2, SU1498 and then exposed to OSMm. *p < 0.05, versus control value, #p < 0.05; versus OSMm value.
